# Supplementary material for: Detection of SARS-CoV-2 Infection in Gargle, Spit, and Sputum Specimens
Source: Microbiol Spectr. 2021 Aug 25;9(1):10.1128/spectrum.00035-21. doi: 10.1128/spectrum.00035-21 (PMC8552728; doi:10.1128/spectrum.00035-21)
Supplement: SUPPLEMENTAL FILE 1 — Supplemental material. Download SPECTRUM00035-21_Supp_1_seq6.pdf, PDF file, 0.4 MB [file spectrum00035-21_supp_1_seq6.pdf]

Detection of SARS-CoV-2 infection in gargle, spit and sputum specimens: Supplementary material

thl

Instructions for sampling gargle, spit and sputum

Gargle sample

- Have a sip of water (1/4 of a glass)
- Gargle
- Spit is back in the container
- Close the container
- Seal it in the zypbag

Spit sample

- Upon waking up, avoid food, water and brushing your teeth until the spit sample is collected
- Repeatedly spit into the container until it is half full with liquid (exclusing bubbles)
- Securely close the container
- Store at room temperature

Sputum sample

- Lean forward and cough hard to get tough mucus from the lower airways.
- Approximately tablespoon of mucus is enough to sample
- Close the container and put the container backto the zypbag seal it icarefully.
- Store the container in the refigerator until the samples are retrieved.

Supplementary appendix 1. Instructions for sampling gargle, spit and sputum collection

	Symptoms experienced since COVID-19 onset N (%)	Symptoms at the time of sample collection N (%)
Any symptoms	31 (86)	24 (67%)
Fever	21 (60)	6 (19)
Sore throat	19 (56)	11 (32)
Cough	27 (79)	15 (44)
Runny nose	22 (65)	12 (34)
Sorthness of breath	5 (16)	2 (6)
Chills	26 (76)	7 (20)
Vomiting	0 (0)	0 (0)
Nausea	6 (17)	3 (9)
Diarrhea	9 (24)	3 (9)
Headache	29 (81)	14 (40)
Neurological signs	2 (6)	1 (3)
Rash	2 (6)	2 (6)
Conjunctivitis	0 (0)	0 (0)
Muscle ache	25 (69)	12 (36)
Joint ache	7 (20)	5 (15)
Loss of appetite	13 (36)	12 (35)
Nose bleeding	0 (0)	0 (0)
Fatigue	31 (86)	25 (71)
Malaise	16 (44)	13 (38)
Seizure	0 (0)	0 (0)
Altered conscious	2 (6)	2 (6)
Anosmia	14 (44)	14 (42)
Other	9 (26)	4 (13)

Supplementary table 1. Symptoms reported by patients
